# Changes in leaf litter decomposition of primary Korean pine forests after degradation succession into secondary broad‐leaved forests

**DOI:** 10.1002/ece3.7903

**Published:** 2021-08-30

**Authors:** Yan‐Mei Fu, Xiu‐Yue Zhang, Dan‐Dan Qi, Fu‐Juan Feng

**Affiliations:** ^1^ School of Life Sciences Northeast Forestry University Harbin China; ^2^ Key Laboratory of Wetland Ecology and Environment Northeast Institute of Geography and Agroecology Chinese Academy Sciences Changchun China

**Keywords:** forest succession, leaf litter decomposition, litter quality, *Pinus koraiensis*, soil microbial community

## Abstract

Forest degradation succession often leads to changes in forest ecosystem functioning. Exactly how the decomposition of leaf litter is affected in a disturbed forest remains unknown. Therefore, in our study, we selected a primary Korean pine forest (PK) and a secondary broad‐leaved forest (SF) affected by clear‐cutting degradation, both in Northeast China. The aim was to explore the response to changes in the leaf litter decomposition converting PK to SF. The mixed litters of PK and SF were decomposed in situ (1 year). The proportion of remaining litter mass, main chemistry, and soil biotic and abiotic factors were assessed during decomposition, and then, we made an in‐depth analysis of the changes in the leaf litter decomposition. According to our results, leaf litter decomposition rate was significantly higher in the PK than that in the SF. Overall, the remaining percent mass of leaf litter's main chemical quality in SF was higher than in PK, indicating that leaf litter chemical turnover in PK was relatively faster. PK had a significantly higher amount of total phospholipid fatty acids (PLFAs) than SF during decomposition. Based on multivariate regression trees, the forest type influenced the soil habitat factors related to leaf litter decomposition more than decomposition time. Structural equation modeling revealed that litter N was strongly and positively affecting litter decomposition, and the changes in actinomycetes PLFA biomass played a more important role among all the functional groups. Selected soil abiotic factors were indirectly driving litter decomposition through coupling with actinomycetes. This study provides evidence for the complex interactions between leaf litter substrate and soil physical–chemical properties in affecting litter decomposition via soil microorganisms.

## Graphical abstract

Leaf litter decay rate significantly decreased after primary Korean pine forests degradation succession into secondary broad‐leaved forests.

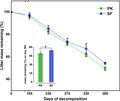

## 1 INTRODUCTION

Leaf litter decomposition is vital for the development of the biogeochemical cycle in nature, so it has become a research focus given its important role in the ecosystem nutrient and carbon cycle (Rovira & Rovira, 2010). Previous research has mainly focused on the biological and abiotic influence on the decomposition of leaf litter and how the interaction of these factors determines litter decay (Allison et al., 2013; Pietsch et al., 2014; Xuluc‐Tolosa et al., 2003). Among these factors, the quality of litter (e.g., litter nitrogen and lignin) and the decomposer community are dominant (He et al., 2016; Mlambo et al., 2019). The study had confirmed that the substrate quality of the litter affects the litter decomposition rate and fundamentally determines the soil nutrient cycle and flux (Peña‐Peña & Irmler, 2016). However, no consistent conclusion regarding this has yet been reached (Dessy et al., 2015; Güsewell & Gessner, 2009). Therefore, performing more detailed research is necessary. Soil environmental conditions are also an important factor affecting leaf litter decomposition (Zhang et al., 2018). Studies have shown that climatic factors (such as moisture and temperature) and soil features (such as pH, soil density, and soil structure) are closely related to leaf litter decomposition because they can affect soil nutrient content and thus further change the reproduction and growth conditions of decomposers (Delgado‐Baquerizo et al., 2015; Valentini et al., 2015). Although most soils contain certain decomposers, the decomposition efficiency of soil microbial communities is different (He et al., 2016; Purahong et al., 2016). In order to clarify the influence of various factors on litter decomposition, we focused on the litter chemistry in different decomposition stages and the microbial composition in the soil in close relation to the decomposition rate, which contributes to the exploration of the factors affecting the litter decomposition against the background of climate change (Bradford et al., 2016; Mayor et al., 2017; Yang et al., 2019). However, the direct and indirect relationships between forest leaf litter and soil remain an open question.

The forests we chose, broad‐leaved and Korean pine mixed forests with Pinus koraiensis, are the most common vegetation growing in the northeastern part of China, also forming the most diverse forest system in this part of the world (Shi et al., 2016). Broad‐leaved Korean pine forests are an important part of the forest in the Northern Hemisphere where the climate is cold, the soil is moist, and islands of permafrost exist. The area belongs to a region sensitive to global climate change (Fu et al., 2019). Early studies have shown that the broad‐leaved Korean pine forests have a strong ability to soil carbon capture (Chen & Li, 2003; Li et al., 2011). As a result of historical factors, the primary Korean pine forests in China have been seriously damaged, with most of them having been disturbed by clear‐cutting. Today, succession has been restored to a large area of middle‐aged and young secondary broad‐leaved forests. The shift of climax communities to secondary broad‐leaved forests reflects degraded succession as a result of human disturbance, which changes the function of forest ecosystems (Antonarakis, 2014). Forest degradation succession leads to gradual changes in stand structure, tree species composition, and internal habitat conditions, as well as the likely inevitable change in leaf litter decomposition characteristics, which is an important factor driving carbon turnover. These changes will provide both positive and negative feedback on forest ecosystem functions. However, systematic researches related to the changes in leaf litter decomposition caused by the degraded succession of Korean pine forests are limited, despite its significance for evaluating the changes in nutrient and carbon sources after forest degradation succession.

Here, we hypothesize that soil habitat conditions (soil physicochemical properties and soil microbial community structure) and leaf litter characteristics change synchronously upon primary Korean pine forest (PK) transition to secondary broad‐leaved forest (SF). We tried to answer the following questions: (a) The differences in the leaf litter decomposition patterns between PK and SF; (b) changes of the soil habitats and the main chemistry (C, N, P, etc.) during litter decomposition; and (c) the degree of the complex relationships between the litter quality characteristics, soil abiotic and biological factors, and leaf litter decomposition.

## 2 MATERIALS AND METHODS

### 2.1 Study area

**FIGURE 1:**
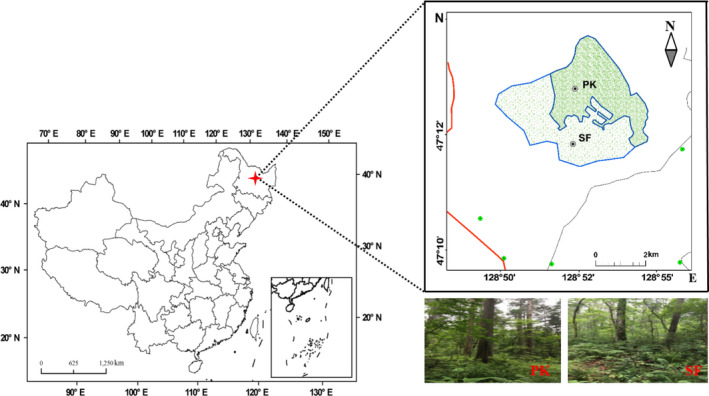
The map of the two areas under research, located in the Liangshui National Natural Reserve in Heilongjiang Province, China. PK, primary Korean pine forest; SF, secondary broad‐leaved forest

This study was conducted in the Liangshui National Natural Reserve (47°12′57″–47°12′49″N, 128°52′17″–128°52′12″E), Heilongjiang, China. The climate is of a continental type with monsoon features. The area enjoys more wind and less rain in the spring. The mean annual precipitation is 676 mm. The snow cover period is 130–180 days. The mean annual temperature of the area is −0.3°C, and the humidity reaches about 78%. For about half of the year, the area is covered by snow. The primary Korean pine forest (PK) and the secondary broad‐leaved forest (SF) were chosen as the study sites (Figure 1). The PK and SF are both dark brown forest soil (Chinese Classification Standard) (Herrmann & Bucksch, 2014), and the color of dry soil is 10YR4/2 (Munsell color chart). The PK and SF cover 11.7 and 9.3 ha of the study area, respectively. Originally, only primary Korean pine forests grew in this area, but part of primary Korean pine forests became bare land in 1961 due to clear‐cutting and was restored to secondary broad‐leaved forest about 60 years later. The two selected forest lands are similar in elevation and slope (Table 1).

**TABLE 1:** Basic information of the two sites in the Liangshui National Natural Reserve in Heilongjiang, China

Forest category	Geographical location	Slope (°)	Altitude (m)	Dominant tree species
PK	47°12′57″N 128°52′17″E	5–6	402	*Pinus koraiensis, Fraxinus mandschurica, Tilia amurensis, Acer mono, Acer ukurunduense, Acer tegmentosum*
SF	47°12′49″N 128°52′12″E	5–6	390	*F. mandschurica, T. amurensis, Phellodendron amurense, A. mono*

### 2.2 Experimental details

We collected the leaf litter between late September 2016 and late October 2016 in the primary Korean pine forest (PK) and secondary broad‐leaved forest (SF). For leaf litter collection (Table 1), ten 2 × 2 m litter collectors were randomly placed in each forest type. At the end of the one‐month collection period, the stored litter in each forest was mixed and the materials were dried at 65°C to constant weight before being placed into litter bags. Some of the obtained materials were analyzed to assess the original contents of the litter, and the remaining material was used for the research regarding decomposition. In early November 2016, geographically and micro‐topographically similar quadrats were selected to circumvent spatial heterogeneity. Three quadrats were established in PK and SF, each with an area of 30 × 30 m, being placed every 20 m in the forest. Nylon bags (20.0 × 15.0 cm with 0.5 mm mesh) were filled with 10 g of the materials mixed beforehand. A total of 200 litter bags were randomly placed in the mineral soil at a depth of 0–10 cm (at about 45° angle relative to the soil surface) in early November 2016 (corresponding to 0 days) in each quadrat. Leaf litter bags were collected (taking 40 bags at a time in each quadrat) in 30 April 2017, 15 June 2017, 31 July 2017, 16 September 2017, and 1 November 2017, corresponding to 185, 230, 275, 320, and 365 days from the starting date. Forty litterbags were harvested and homogeneously mixed to create one sample per quadrat at each sample time in PK and SF, respectively (*n* = 3). Thus, the mixed materials from each quadrat were dried at 65°C to constant mass. The average dry weight of litter bags in each quadrat was calculated for measurement of leaf litter decomposition rate, and the subsamples were milled and then stored at room temperature for assay of the leaf litter quality.

When collecting litterbags from each quadrat in the PK and SF, the soil samples were taken from next to the litter bag at the same time. Soil samples refer to litter samples. Thereafter, the composite soil was filtered through 2‐mm mesh and then divided into two groups of subsamples. We froze the first subsample to study the microbial properties, and the other one was fully air‐dried for at least 1 week and then used to determine the physical and chemical properties of soil.

### 2.3 Analysis methods

#### 2.3.1 Leaf litter chemical analysis

Leaf litter C and N contents were studied using a CHNOS Elemental Analyzer (Elemental Analyzer, Elementar Analysensysteme GmbH). Leaf litter P and K were studied with an inductively coupled plasma optical emission spectrometer (ICAP 6300 ICP‐OES spectrometer, Thermo Scientific, Thermo Electron Corporation). Leaf litter lignin and cellulose contents were measured according to the acid‐detergent lignin (Wei et al., 2016). This method yields cellulose and acid‐detergent lignin, which has been referred to as AUR (acid unhydrolyzable residue), by calculating the weight remaining after washing litter with different reagents.

#### 2.3.2 Soil physicochemical properties measurements

Soil temperature of the topsoil (0–10 cm) (T) was calculated with an electronic thermometer, and soil bulk density (BD) was calculated using a soil‐cutting ring (100 cm^3^). Soil organic carbon (SOC) (1 g) was analyzed with the oxidation method with potassium dichromate, and the mass was determined using a visible spectrophotometer (JH‐14‐08 723). Soil total nitrogen (STN) (1 g) was determined by applying an element determiner (EuroEA3000, Elementar). Soil available phosphorus (SAP) (5 g) was calculated through Bray‐1P extraction, and colorimetric analysis was performed using a molybdenum blue process. Soil available potassium (SAK) (5 g) was separated using NH_4_OAc and determined using a flame parameter. Soil water content (SWC) (10 g) was calculated through heating and calculating the weight of the lost materials, and the soil pH was determined using a pH meter after the earth had been deprived of water.

#### 2.3.3 Soil microbial community

One of the subsamples was used to examine the microbes growing in the areas by analyzing the phospholipid fatty acids (PLFAs) (Sandoval et al., 2013). 5 g of lyophilized soil was separated from the areas using the Bligh and Dyer method (White et al., 1997) to check the individual fatty acids in the soil with the help of gas chromatography and mass spectrometry. The peaks were calculated based on the standard of 19:0 and through the qualitative mix of bacterial fatty acid methyl esters. We used nmol/g dry soil to measure fatty acids. We used a combination of general bacteria (Bac), Gram‐positive bacteria (G+), Gram‐negative bacteria (G−), actinomycetes (Act), and fungi (Fun) to examine the microbial populations. The PLFAs consisted of Bac (Carney et al., 2007; Frostegård & Bååth, 1996), sum of i15:0 + a15:0 + i16:0 + i15:0 2OH + 16:1ω7c + 16:1ω9c + 16:1 ω5c + 10Me 16:0 + i17:0 + a17:0 + cy17:0 + 17:0 + 18:1ω7c + 18:1ω7 t + 18:1ω5c + 10Me 18:0; Fun (Bossio & Scow, 1998), 18:2ω6; G+ (Berry, 1989), sum of i15:0, a15:0, i16:0, i17:0, a17:0; FB, Fun/Bac; G− (Moore‐Kucera & Dick, 2008), sum of cy17:0, cy19:0, 16:1ω7,16:1ω9, 18:1ω7, 17:1ω8, 17:1ω9; Act (Moore‐Kucera & Dick, 2008), sum of 10Me16:0, 10Me17:0, 10Me18:0.

### 2.4 Estimation of forest biomass

Forest biomass is the functional index of a forest ecosystem and an important index of forest type (Raich & Nadelhoffer, 1989). At present, relative growth models are well‐accepted for estimating tree biomass (Yang & Fan, 2011). In the summer of 2016, we measured the diameter at breast height (D) of standing living trees larger than 5cm in each quadrat. Based on the expression of conventional biomass model (Table A1) (Zhang et al., 2010), we calculated the tree species biomass in each quadrat, and the biomass of the PK and SF was cumulative gain (the total biomass of the quadrat tree species/100 m^2^ × the study area).

### 2.5 Calculations

The calculations for leaf litter decomposition rate (as percent (%) mass remaining), chemical mass remaining (%) (Zhang et al., 2018), the leaf litter decomposition rate constant (*k*), and the time needed to reach 95% mass loss were calculated as:

(1) $$\text{Mass remaining}\;\left(\%\right)=\frac{W_{t}}{W_{0}}\times 100\%$$

(2) $$\text{Chemical mass remaining}\;\left(\%\right)=\frac{C_{t}W_{t}}{C_{0}W_{0}}\times 100\%$$

(3) $$W_{t}/W_{0}=e^{‐kt}$$

(4) $$T_{0.95}=\frac{\ln0.05}{\left(‐k\right)}$$

where *W*_0_ is the original weight of the leaf litter (10 g in the research), *W_t_* is leaf litter residual mass (g), *C*_0_ is the original concentration of the chemical, and *C_t_* is the concentration of the chemical after time *t*. The leaf litter decomposition rate constant (*k*) was calculated using *Olson's* decay model (Olson, 1963). *T*_0.95_ is the time needed to reach 95% mass loss.

### 2.6 Statistical analysis

The normality and heteroscedasticity of the data diversity were examined using the Kolmogorov–Smirnov test and Levene's test, respectively. The *k* values between PK and SF were determined through an independent‐sample *t* test. The diversity in physicochemical properties, soil microbial properties, and leaf litter quality was analyzed using analysis of variance (ANOVA). The relative importance of forest type and decomposition time to the change in soil habitat factors in general (soil physicochemical properties and soil microbial community) was quantitatively analyzed using the multivariate regression trees (MRT) method. The means of the MRT analysis were set using the R Package “mvpart” (Ye et al., 2019).

**FIGURE A1:**
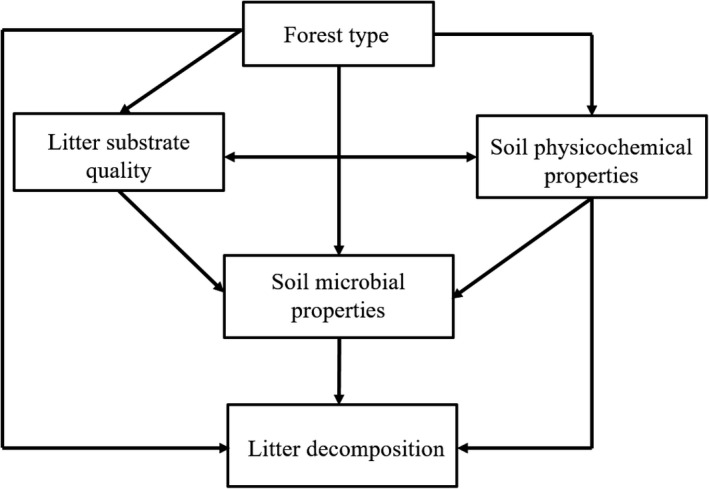
Conceptual framework of expected causality between forest type and litter decomposition in a priori structural equation model (Wang et al., 2014)

The data could not meet the linear relationship through the ordinary log transformation, so we used the generalized ADDITIVE models (GAM) in the “mgcv” package to evaluate and convert the nonlinear relationship between leaf litter substrate, soil physicochemical, and soil microbial PLFA biomass. All linear model fits were evaluated by adopting normality and heteroscedasticity, and the result was log, log + 1, square root, or power three, converted if needed (Wang, He, et al., 2018; Wang, Kwak, et al., 2018) (Table A2). We have built the full model, including all factors transformed by GAM. Then using stepwise regression based on the GAM to eliminate the multicollinearity factor (AIC and BIC rules are considered simultaneously) (Harrell, 2001). The structural equation model (SEM) selected was adopted to determine the complicated relationship between soil habitat factors and leaf litter decomposition based on AIC. The structural equation model (SEM) selected was adopted to determine the complicated relationship between soil habitat factors and leaf litter decomposition. SEM was based on the relationships between soil physical and chemical properties, soil microbial community structure, leaf litter quality, and leaf litter decomposition according to the conceptual model (Figure A1). We included the remaining factors after excluding collinearity into the structural equation model by stepwise regression, including five endogenous variables, litter N, actinomycetes, log(pH), SAK, SWC. To obtain the most parsimonious model, nonsignificant pathways were eliminated, and a new variable, forest type (characterized by forest biomass), was introduced. SEM was able to delineate the patterns of the direct and indirect effects of explanatory variables by formulating a causal model based on the data of this study. For the analysis, we employed robust maximum‐likelihood estimation procedures, for example, the *p*‐value, the chi‐square test, comparative fit index (CFI), and goodness of fit (GFI). The statistical significance was the 5% level. The statistical analyses were performed with the R software (version 3.5.2), and SEM was analyzed using AMOS version 7.0 (IBM SPSS Inc.).

## 3 RESULTS

### 3.1 Changes of abiotic and biotic factors in the soil

From the beginning of decomposition (1 November 2016) to the end of decomposition (1 November 2017), the mean values of T, BD, SWC, STN, and SAK in PK were all significantly higher than those of SF, except SOC, and the mean values of pH and SAP were not significantly different between forest types (Table 2). The two‐way ANOVA showed that forest type, season, and their interaction significantly affected T, BD, SWC, pH, SAP, SOC, STN, and SAK (Table A3).

**TABLE 2:** Comparison of the average soil characteristics value of PK and SF during litter decomposition

Forest type	T (°C)	BD (g/cm)	SWC (%)	pH	SOC (g/kg)	STN (g/kg)	SAP (mg/kg)	SAK (mg/kg)
PK	12.27	1.24	38.86	5.39	124.97	27.63	13.12	576.76
	(0.19)	(0.03)	(0.77)	(0.06)	(21.00)	(0.26)	(0.29)	(23.85)
SF	9.39	1.07	31.25	5.32	181.91	27.14	12.91	482.88
	(0.72)	(0.05)	(1.56)	(0.03)	(26.72)	(0.09)	(0.16)	(24.28)
*F*	44.30	25.03	58.12	3.79	8.42	9.61	1.20	22.72
*p*	**	**	**	ns	*	*	ns	**

Note

*p < .05; **p < .01; values are represented as means ± SE.

Abbreviation: ns, not significant.

**FIGURE 2:**
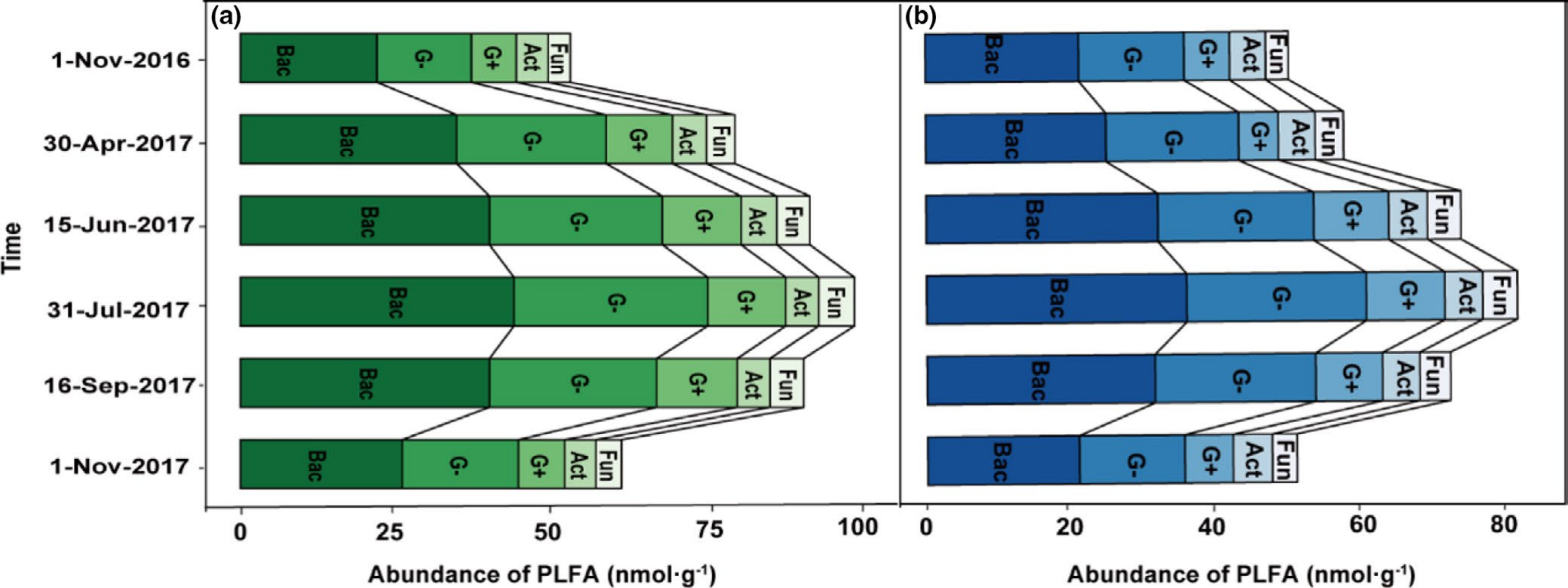
Seasonal dynamics in microbial community abundance in situ in the leaf litter decomposition period in the PK (a) and (b) SF soils. Lines represent the component connection line between groups

At the beginning (from 1 November 2016 to 30 April 2017) and end of decomposition (from 16 September 2017 to 1 November 2017), we found an obvious trend in total PLFA, bacteria (Bac), fungi (Fun), G+, G−, and actinomycetes (Act) PLFA biomass between two forest types (Figure 2). During the whole experiment period, the average PLFA biomass of Bac, G+, G−, Fun, and Act in SF was significantly lower than in PK (*p* < .05) (Table A4). Compared with PK, the total PLFA biomass of the SF decreased by 15.41%. We also found that the bacterial community accounted for the largest proportion and fungi for the smallest proportion during litter decomposition (Figure 2). Except for G+, the rest of the microbial communities' PLFA biomass reached the maximum on 31 July 2017 (Figure 2a,b).

### 3.2 Leaf litter mass remaining dynamics

**FIGURE 3:**
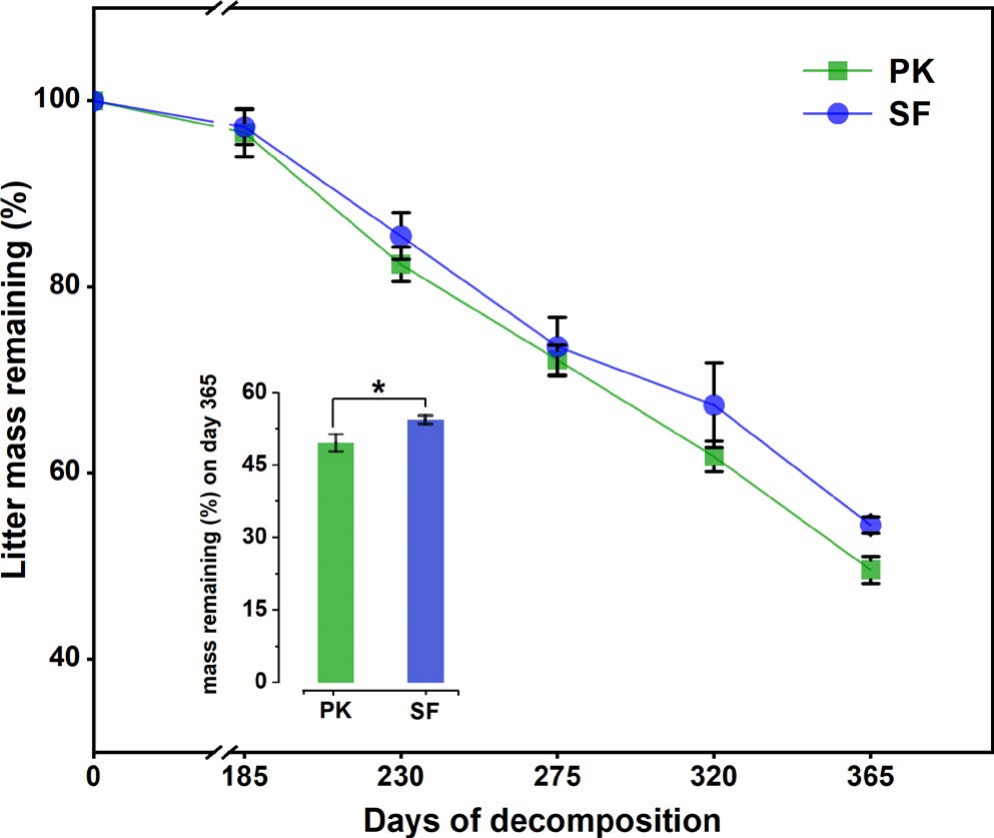
Average percent of leaf litter remaining mass (means ± SE) between PK and SF. Note: a significant difference between PK and SF is indicated by * (*p* < .05)

Litter remaining mass showed a generally consistent trend in PK and SF (Figure 3). Leaf litter decomposition was relatively slow before 185 days, speeding up thereafter. After 365 days of decomposition, the litter mass remaining (%) in SF was significantly higher than in PK. Similarly, we analyzed the mass loss patterns during decomposition based on the average decomposition rate constant (*k*) using Olson's decay model. The results showed that PK had a significantly higher *k* value (0.656 year^−1^) than SF (0.589 year^−1^), and according to the calculation, after 4.566 years, the PK leaf litter decomposition would reach 95%. SF would require 5.088 years to achieve the same result. The data illustrated that leaf litter in PK decomposed more easily than those in SF during the research period (Table 3).

**TABLE 3:** *Olson's* exponential regression equations for leaf litter decomposition rates in PK and SF

Forest type	Regression model	*R* ^2^	*T*_95%_ (year)
PK	*y* = 1.134 × 10^0.656t^	0.703	4.566 ± 0.100a
SF	*y* = 1.124 × 10^0.589t^	0.700	5.088 ± 0.182b

Note

*T*_95%_ is the time required for 95% mass loss. The lowercase letters show that the data between forest types are significant (*p* < .05).

### 3.3 Shifts in litter chemical mass remaining

**FIGURE 4:**
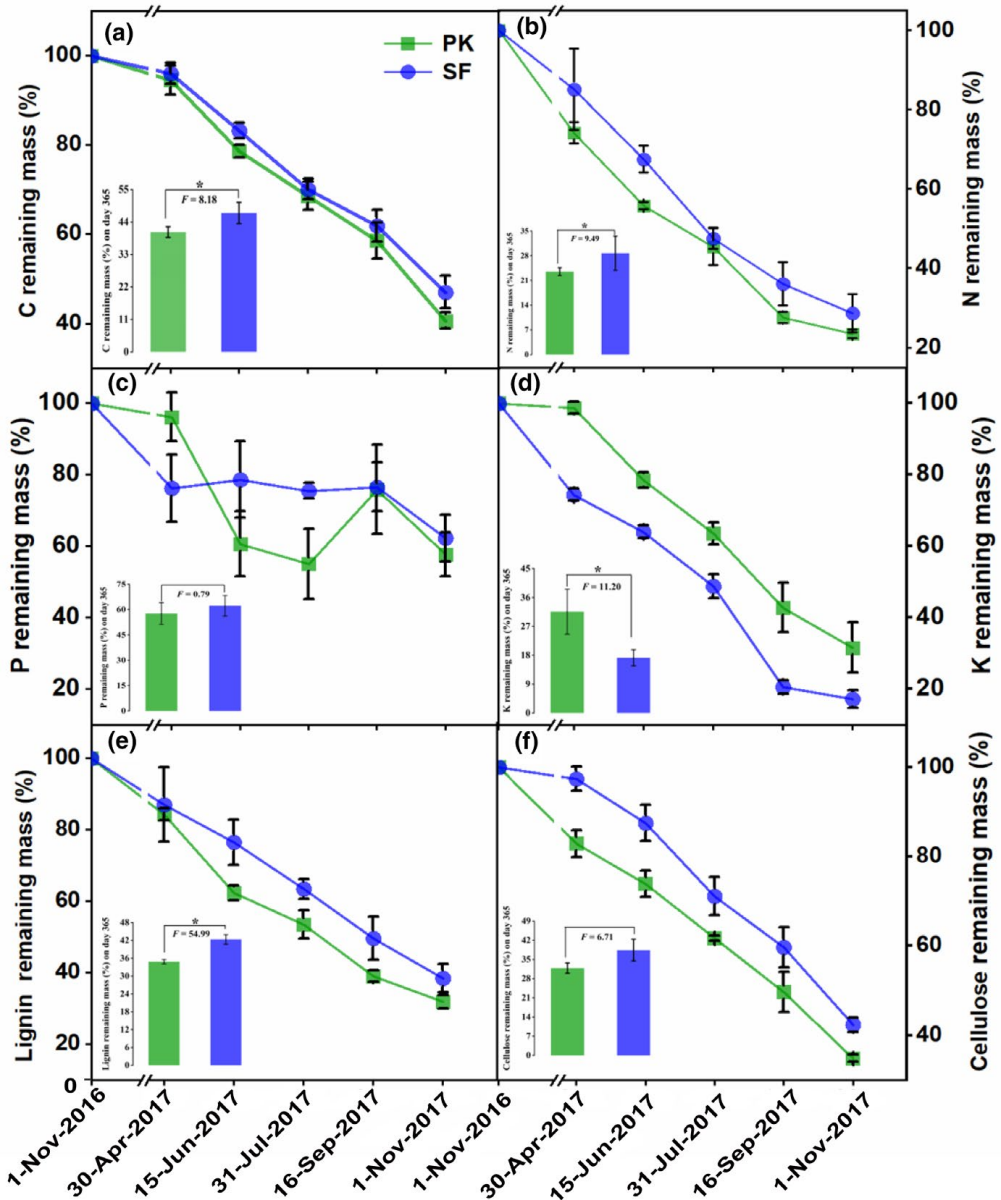
Percent (%) remaining mass of (a) C, (b) N, (c) P, (d) K, (e) lignin, and (f) cellulose in decomposing leaf litter in PK and SF. The column charts represent the remaining mass (%) of litter in various chemistry at 365 days of decomposition. Note: a significant difference between PK and SF at 365 days of decomposition is indicated by * (*p* < .05); otherwise, it is not marked. Statistics are illustrated through means ± SE. The breakpoint on the x‐axis represents the frost period

We could see the remaining mass (%) of litter in various chemistry during decomposition by Figure 4. Direct release of C, K, lignin, and cellulose occurred through the whole decomposition period (Figure 4a,d–f). N showed slight net immobilization after 320 days of decomposition (Figure 4b), whereas P was enriched in varying degrees during different stages of decomposition (Figure 4c). After 1 year, the proportions of P remaining in the initial litter were within the range of 57.71%–62.26%. The proportions of N and K remaining in the initial litter were within 23.52%–28.73% and 17.24%–31.60%, respectively. The results showed that N and K had higher release rates than P. Although some differences were observed in the temporal of release and immobilization phase in the two forest types compared with the initial content, the final content showed a downward trend. Compared with PK, the remaining C, N, and lignin (%) in SF (*p* < .05) were significantly higher on the 350 days of decomposition (the remaining P and cellulose no significant). The remaining K (%) was significantly higher in PK than SF (*p* < .05).

### 3.4 Factors driving leaf litter decomposition after the degradation of primary Korean pine forests

**FIGURE 5:**
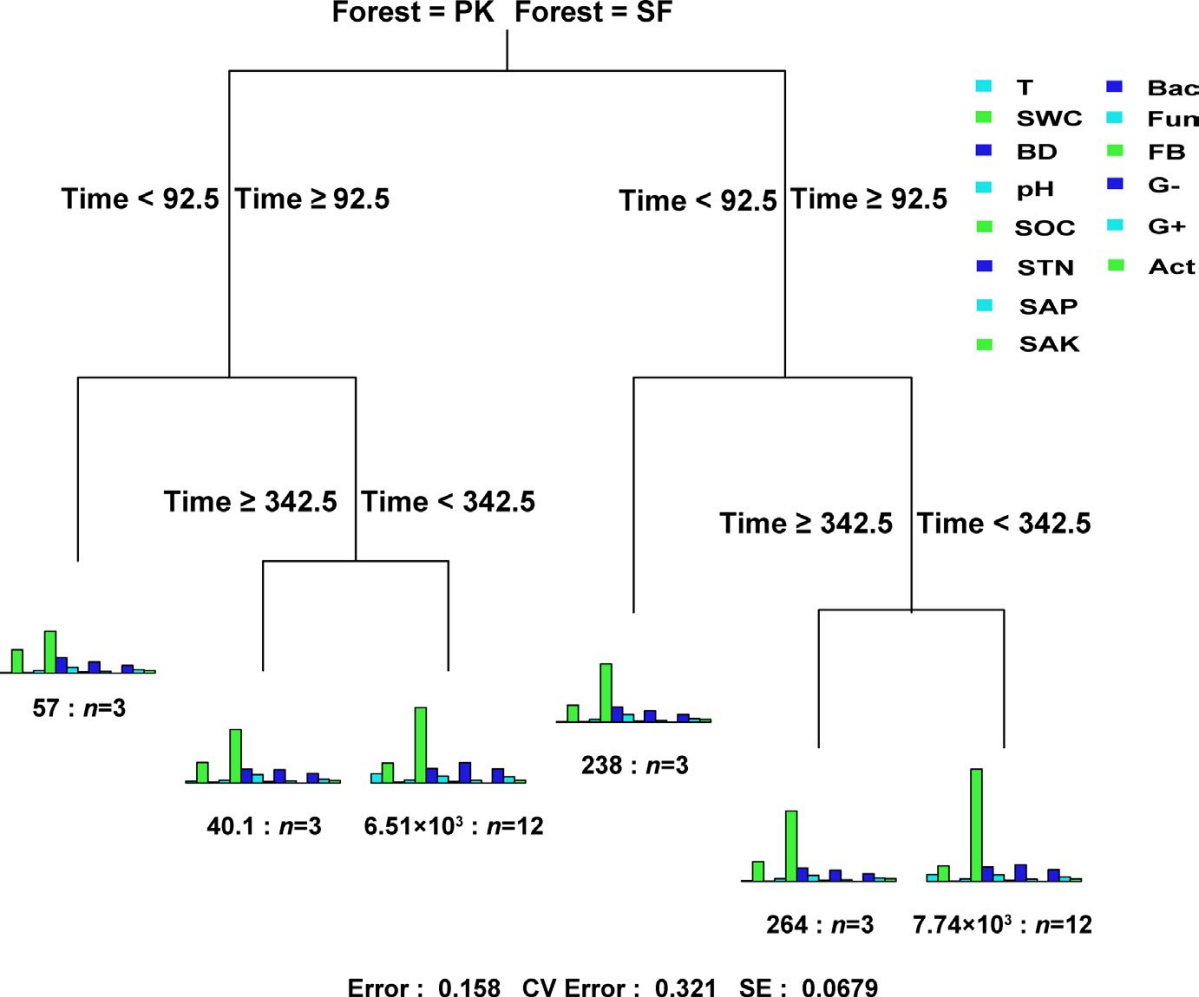
Multivariate regression tree analysis of soil habitat factors, forest types, and decomposition time. The different colors in each column represent different soil properties. Time represents decomposition days

**FIGURE 6:**
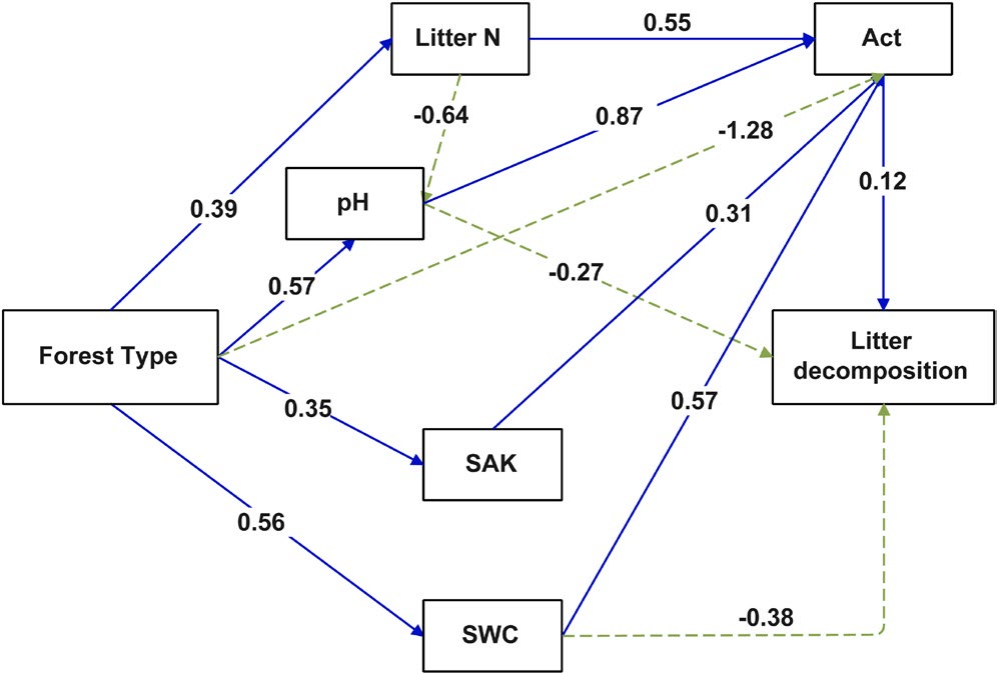
Models illustrating both the direct and indirect influence of factors on the litter decomposition between PK and SF. The blue solid lines represent the paths that positively impacted the leaf litter decomposition, while the green dotted lines represent paths that had negative impacts (*p* = .91, *χ* ^2^ (CMIN)/degrees of freedom (DF) = 1.90, goodness of fit (GFI) = 0.93, comparative fit index (CFI) = 0.97)

The relative importance of the two non‐numerical variables (forest type and decomposition time) on soil habitat factors related to litter decomposition was analyzed using the MRT method. According to Figure 5, the first segmentation was taken using forest type as the node, indicating that the effect of forest type on soil habitat was more important, and forest type and decomposition time could explain 84.2% of the variance in soil habitat. Then, we identified the significant main factors affecting leaf litter decomposition by using stepwise regression. They were litter N content, soil physicochemical indexes (soil pH, SWC, and SAK), and soil actinomycetes PLFA biomass (Table A5). To continue examining the complex relationships between the litter quality characteristics, soil abiotic and biological factors, and leaf litter decomposition, we used the SEM with the selected key drivers (Figure 6). The results showed that the forest type directly influenced the litter N, pH, SWC, and SAK. In addition, pH, SWC, and actinomycetes were directly driving leaf litter decomposition, and eventually, litter N, pH, SWC, and SAK indirectly affected leaf litter decomposition by affecting Act. Of which, the total effects of the litter N (0.17) and actinomycetes (0.12) were the strongly and positively affecting litter decomposition, and the total effects of SWC were the most important negative factor affecting litter decomposition (−0.31).

## 4 DISCUSSION

### 4.1 Diversification of leaf litter decomposition after the degradation succession of primary Korean pine forests

Many studies have shown that forest degradation leads to several changes in soil habitat, which likely affect leaf litter decomposition (Lecerf & Richardson, 2010; Raich & Nadelhoffer, 1989). In this study, with the change of soil habitat conditions (Table 1 and Figure 2), the decomposition rate of leaf litter significantly decreased after primary Korean pine forest degradation succession into secondary broad‐leaved forests (Figure 3 and Table 3). For a variety of interference modes, most studies support the conclusion that litter degrades significantly faster in undisturbed forests compared with disturbed forests and old‐growth forests are more efficient at nutrient cycling (Brown & Lugo, 1990; Chazdon et al., 2010). For example, Blair and Crossley (1988) found that the leaf litter decomposition rate in forests in southeastern United States that had been subjected to clear‐cutting significantly declined. Blanco et al. (2011) explored the effects of thinning on the decomposition of forest litter in the Pyrenees, they also found that whatever the intensity of thinning, it would slow down its decomposition. These results were consistent with our findings.

Leaf litter decomposition could return nutrients to the soil, completing the biological cycle of nutrients in the ecosystem and providing necessary elements for forest growth, which is an important mechanism of forest self‐fertilization (Manzoni et al., 2010; Wang et al., 2019). Ge et al. (2019) indicated that the related transformation and decomposition features of leaf litter nutrients result in corresponding changes in soil fertility. Dent et al. (2006) found that an increase in soil fertility was basically synchronized with an increase in the leaf litter decomposition rate. Our study indicated that not only the leaf litter decomposition rate constant (*k*) decreased significantly, but also the remaining mass (%) of the litter main chemistry (C, N, and lignin) in the primary Korean pine forest was lower than that of the secondary broad‐leaved forest during decomposition. The results reflected that the turnover of leaf litter chemistry in the primary Korean pine forest was relatively faster in general during decomposition (Figure 4a,b,e), so that it may more quickly and effectively replenish the nutrients consumed by the plants in the soil, thereby improving the material circulation speed within the whole community (Dent et al., 2006; Kotowska et al., 2015). Temperate forest ecosystems, including primary Korean pine forests, generally have the phenomenon of slow nitrogen turnover and relative lack of soil nitrogen (Chen & Li, 2003). Nitrogen is considered to be one of the most important factors limiting plant growth in a forest ecosystem, which has an important impact on forest community succession (Frazer et al., 1990). Generally, the main sources of N in forest soil are the return of litter and microbial decomposition (Delgado‐Baquerizo et al., 2015). Therefore, compared with the primary Korean pine forest (Figure 4b), the secondary broad‐leaved forest had a relatively weak ability to release nitrogen into soil through leaf litter decomposition which may aggravate the phenomenon of “nitrogen limitation.” Nitrogen limitation inhibited microbial activity and growth and also can form negative feedback on plant growth and ecosystem structure and function (Schmidt et al., 2016), so this may be one of the reasons for the significant decrease of the decomposition rate constant (*k*) after the degradation succession of primary Korean pine forests. And if this change continues for a longer time scale, it will increase the difficulty for the secondary forest to return to the original vegetation type (primary Korean pine broad‐leaved forest).

### 4.2 Factors controlling leaf litter decomposition alterations based on SEM

In different periods of forest succession, profound changes in soil habitat caused by differences in aboveground vegetation affect the leaf litter decomposition (Forest et al., 2013; Prescott et al., 2000). Previous research demonstrated that leaf litter decomposition may be influenced by litter quality, soil physicochemical properties, and/or biological factor adjustments (Bray et al., 2012; García‐Palacios et al., 2016; Horodecki & Jagodziński, 2019; Polyakova & Billor, 2008). In this study, we found that leaf litter decomposition was significantly different in the primary Korean pine forest and secondary broad‐leaved forest. The variations were apparently related to differences between the forest types in litter quality and soil abiotic and biological properties (Figures 5 and 6). The result was similar to that of most other studies (Bhatnagar et al., 2018; Keiser et al., 2013; Sariyildiz et al., 2005).

Litter substrate quality exerts a great influence on litter decomposition (Aerts, 1997; Wang, He, et al., 2018; Wang, Kwak, et al., 2018). Litter N content strongly and positively influenced the leaf litter decomposition that has changed after degradation succession of primary Korean pine forest, being similar to most research results (Horodecki & Jagodziński, 2017; Lin et al., 2018; Shan & Jian, 2012). One reason for this finding is that nitrogenous substances are mostly unstable compounds (Zhang et al., 2018), and thus, their solubility in water is usually higher. Most water‐soluble compounds are easily decomposed by microorganisms (Güsewell & Verhoeven, 2006), contributing to the decomposition. Another reason for decomposition may be the nitrogen constraints in disturbed and restored forests. The availability of nitrogen may be one of the main factors limiting forest recovery as a result of its interfering with forest leaf litter decomposition (Mo et al., 2006), which was supported by the SEM in our research (Figure 6). Furthermore, compared with the secondary broad‐leaved forest, a higher content of lignin was found in the leaf litter of the primary Korean pine forest, but unexpectedly, lignin did not become a limiting factor affecting litter decomposition. The reason for this may be due to the nonadditive effect in the decomposition process of mixed litter (Yinong et al., 2016). Environmental factors are usually the main force driving decomposition because the activities of decomposers are often affected by SWC, pH, T, BD, etc. (Peña‐Peña & Irmler, 2016; Wall et al., 2008). Through stepwise regression, the important abiotic factors selected in this study were SWC, pH, and SAK (Table A5). We further discovered in the SEM that these factors could indirectly affect the leaf litter decomposition process by coupling with actinomycetes. Some studies have suggested that soil microbes and litter decomposition are part of the same continuum (Krumins et al., 2010; Ren et al., 2017). Seasonal dynamics of actinomycetes PLFA biomass during litter decomposition were found in the SEM to considerably positively influence the leaf litter decomposition compared with other microbial groups (Figure 6). Previous studies had shown that actinomycetes could promote litter decomposition (Allison et al., 2013; Johansson et al., 2010). In our study, the average PLFAs biomass of actinomycetes was significantly higher in the primary Korean pine forests than secondary broad‐leaved forests during decomposition. It may be another reason that could explain the decomposition rate constant (*k*) of secondary broad‐leaved forest was significantly lower than that of primary Korean pine forest except for nitrogen limitation. Helfrich et al. (2015) further found that different microbial groups had different responses to litter decomposition, and among them, soil actinomycetes were very sensitive and they even considered that actinomycetes could replace soil fungi to play a role in the decomposition of soil organic material. However, different groups of microbial communities have different contributions to the litter decomposition. Therefore, we strongly recommend that future in‐depth studies primarily focus on how functional microorganisms affect leaf litter decomposition.

## 5 CONCLUSIONS

In this study, we demonstrated that leaf litter decomposition was significantly different between the PK and SF. Then, we analyzed the main factors that caused changes in leaf litter decomposition. The results showed that decomposition rate constant (*k*) in the PK was significantly higher than SF and the turnover time of the main nutrient elements (C and N) in the leaf litter in SF was significantly longer than that of PK. MRT analysis indicating that the effect of forest type on soil habitat was more important than decomposition time. In addition, SEM showed that pH, SWC, and actinomycetes were directly driving leaf litter decomposition, and eventually, leaf litter N, pH, SAK, and SWC could indirectly affect leaf litter decomposition by affecting actinomycetes. Of which, the leaf litter N had the strongly and positively affecting decomposition and changes in the actinomycetes PLFA biomass was more important than changes in the other functional groups in terms of affecting litter decomposition. In the case of a sharp decline in the primary Korean pine forest, quantifying these mechanisms is crucial to accurately integrate litter decomposition into the ecosystem carbon dynamics of the primary Korean pine forest.

## CONFLICT OF INTEREST

The authors declare no conflict of interest.

## AUTHOR CONTRIBUTIONS

**Yan‐Mei Fu:** Conceptualization (supporting); Data curation (equal); Formal analysis (equal); Investigation (lead); Methodology (lead); Resources (equal); Software (equal); Writing‐original draft (lead); Writing‐review & editing (lead). **Xiu‐Yue Zhang:** Methodology (supporting); Writing‐original draft (supporting); Writing‐review & editing (supporting). **Dan‐Dan Qi:** Conceptualization (supporting); Methodology (supporting); Software (equal). **Fu‐Juan Feng:** Conceptualization (lead); Formal analysis (equal); Funding acquisition (lead); Methodology (equal); Writing‐review & editing (supporting).

### OPEN RESEARCH BADGES

This article has earned an Open Data Badge for making publicly available the digitally‐shareable data necessary to reproduce the reported results. The data is available at https://doi.org/10.5061/dryad.b5mkkwhdh.

## Data Availability

Data are available at https://datadryad.org/stash/resources/126965/review (https://doi.org/10.5061/dryad.b5mkkwhdh).

## APPENDIX A

**TABLE A1:** Expression of conventional biomass model for the species in the study site

Species	Biomass equation	PK forest biomass (t/ha)	SF forest biomass (t/ha)
*Pinus koraiensis*	$W=0.063D^{2.521}$	232.16 ± 23.47a	178.43 ± 21.08b
*Phellodendron amurense*, *Fraxinus mandschurica*	$W=101.871+2.468\times \log10^{D}$		
*Tilia amurensis*	$W=0.055D^{2.360}$		
*Acer tegmentosum*, *Acer ukurunduense*, *Acer mono*	$W=1.939D^{1.648}$		

Note

The letters show that the data between forest types are significant (*p* < .05).

Abbreviations: *D*, diameter at breast height; *W*, tree species biomass.

**TABLE A2:** Linear relationships between litter decomposition and leaf litter substrate properties, soil physical–chemical, and microbial community structure by transferring forms

Category	Indexes	Decomposition
		Function
Leaf litter substrate properties	C	C
	**N**	**N + sqrt (N)**
	**P**	**P + P^2^**
	**K**	**K + log K**
	**Cellulose**	**Cellulose + **$\sqrt{\mathrm{Cellulose}}$ + **log (Cellulose)**
	**Lignin**	**Lignin + log (Lignin)**
Soil physical–chemical properties	SOC	SOC
	STN	STN
	**SAP**	**SAP + log (SAP) + SAP^2^ + SAP^3^**
	**SAK**	**SAK + log (SAK)**
	BD	BD
	**pH**	**pH + log(pH)**
	T	T
	SWC	SWC
Soil microbial properties	Bac	Bac
	Fun	Fun
	FB	FB
	G+	G+
	G−	G−
	Act	Act

Note

All the nonlinear functions of factors were transferred to linear based on general addition model using R 3.5.2.

Abbreviations: Act, actinomycetes; Bac, bacteria; BD, soil bulk density; C, leaf litter carbon; Cellulose, leaf litter cellulose; FB, fungi/bacteria; Fun, fungi; G−, gram‐negative bacteria; G+, gram‐positive bacteria; K, leaf litter potassium; Lignin, leaf litter lignin; N, leaf litter nitrogen; P, leaf litter phosphorus; pH, soil pH; SAK, soil available potassium; SAP, soil available phosphorus; SOC, soil organic carbon; STN, soil total nitrogen; SWC, soil water content; T, soil temperature.

The part in bold indicates that the conversion is based on GAM.

**TABLE A3:** The effects of forest type (F) and season (S) on the soil physicochemical properties

*F* (*p*) value	T	BD	SWC	pH	SAP	SOC	STN	SAK
F	77.04 (***)	32.02 (***)	129.62 (***)	16.07 (**)	41.23 (***)	405.74 (***)	195.00 (***)	53.71 (***)
S	264.11 (***)	13.16 (***)	29.71 (***)	62.94 (***)	49.61 (***)	122.23 (***)	45.30 (***)	40.74 (***)
F × S	4.72 (**)	16.99 (***)	21.47 (***)	42.59 (***)	3.53 (*)	32.76 (***)	18.83 (***)	31.38 (***)

Note

***, **, and * indicate significant differences at *p* < .001, *p* < .01, and *p* < .05, respectively.

Abbreviations: BD, soil bulk density; pH, soil pH; SAK, soil available potassium; SAP, soil available phosphorus; SOC, soil organic carbon; STN, soil total nitrogen; SWC, soil water content; T, soil temperature.

**TABLE A4:** The soil microbial PLFA biomass (nmol/g) in the primary Korean pine forest (PK) and secondary broad‐leaved forest (SF)

Forest type	Total PLFAs	Bac	Fun	G+	G−	Act
PK	464.74 ± 19.36	33.39 ± 8.56	4.65 ± 0.83	10.25 ± 2.67	23.19 ± 5.89	5.84 ± 1.53
SF	393.14 ± 16.02	28.33 ± 6.48	4.10 ± 0.68	8.31 ± 2.43	19.74 ± 4.35	4.89 ± 1.15
*F*	24.35	16.12	32.42	24.91	31.23	22.79
*p*	**	*	**	**	**	**

Note

**p* < .05; ***p* < .01.

Abbreviations: Act, actinomycetes; Bac, bacteria; Fun, fungi; G−, gram‐negative bacteria; G+, gram‐positive bacteria; ns, not significant.

**TABLE A5:** Results of the stepwise regression between leaf litter decomposition and leaf litter substrate, soil physicochemical, and soil microbial community

Model (Standardization coefficient)	Adjusted *R* ^2^	*F*	*p*	AIC/BIC
0.853*STN	0.720	90.988	***	100.734/105.484
0.862*STN−0.208*SWC	0.757	55.664	***	96.480/102.814
0.556*STN−0.370*SWC+0.435*N	0.821	54.680	***	86.348/94.265
0.374*STN−0.447*SWC+0.497*N−0.221*log(pH)	0.844	48.214	***	82.428/91.929
0.060*STN−0.552*SWC+0.753*N−0.440*log(pH)+0.287*Act	0.889	56.875	***	71.022/82.107
−0.573*SWC+0.796*N−0.472*log(pH)+0.307*Act	0.892	72.964	***	69.245/78.746
**0.795*N**−**0**.**596*SWC**−**0.540*log (pH)+0.129*SAK+0.304 *Act**	**0.902**	**65.427**	*******	**66.432/77.516**

Note

****p* < .001.

Abbreviations: Act, actinomycetes; N, leaf litter nitrogen; pH, soil pH; SAK, soil available potassium; STN, soil total nitrogen; SWC, soil water content.

The part in bold indicates that the conversion is based on GAM.
